# Generator of Slow Denial-of-Service Cyber Attacks †

**DOI:** 10.3390/s21165473

**Published:** 2021-08-13

**Authors:** Marek Sikora, Radek Fujdiak, Karel Kuchar, Eva Holasova, Jiri Misurec

**Affiliations:** Department of Telecommunications, Faculty of Electrical Engineering and Communications, Brno University of Technology, Technicka 12, 616 00 Brno, Czech Republic; fujdiak@feec.vutbr.cz (R.F.); xkucha24@stud.feec.vutbr.cz (K.K.); xholas08@stud.feec.vutbr.cz (E.H.); misurec@feec.vutbr.cz (J.M.)

**Keywords:** network security, slow DoS attacks, vulnerability testing, prevention, detection

## Abstract

In today’s world, the volume of cyber attacks grows every year. These attacks can cause many people or companies high financial losses or loss of private data. One of the most common types of attack on the Internet is a DoS (denial-of-service) attack, which, despite its simplicity, can cause catastrophic consequences. A slow DoS attack attempts to make the Internet service unavailable to users. Due to the small data flows, these attacks are very similar to legitimate users with a slow Internet connection. Accurate detection of these attacks is one of the biggest challenges in cybersecurity. In this paper, we implemented our proposal of eleven major and most dangerous slow DoS attacks and introduced an advanced attack generator for testing vulnerabilities of protocols, servers, and services. The main motivation for this research was the absence of a similarly comprehensive generator for testing slow DoS vulnerabilities in network systems. We built an experimental environment for testing our generator, and then we performed a security analysis of the five most used web servers. Based on the discovered vulnerabilities, we also discuss preventive and detection techniques to mitigate the attacks. In future research, our generator can be used for testing slow DoS security vulnerabilities and increasing the level of cyber security of various network systems.

## 1. Introduction

Nowadays, with the ever-growing number of Internet users and the expanding range of Internet services, the demands on the security of users’ data, services, and privacy are also growing. One type of cyber attack is the DoS (denial-of-service) attack. The main goal of this attack is to make the target Internet service unavailable to other users, or, at least, to degrade the quality and speed of the service. Most often, DoS attacks are targeted on web servers to prevent users from accessing web content. The primary targets of DoS attacks include web, mail, database, file, and domain system servers, and remote access services. Attacks can also block traffic on the target internal networks [1].

DoS attacks have gradually evolved from primitive flood attacks to the current sophisticated attacks targeting application protocols of the TCP/IP (Transmission Control Protocol/Internet Protocol) model, especially the HTTP (Hypertext Transfer Protocol) [2]. These attacks communicate validly on lower-layer protocols. This makes it challenging for many detection mechanisms to distinguish them from legitimate traffic. One of these attacks are slow DoS attacks. Their main characteristic is a very slow data flow. Due to unhandled system vulnerabilities or abnormal service usage, these attacks can overload the target server and cause a denial of service to other users with just a few packets. Due to low data flows and usually valid use of all protocols, these attacks are very similar to legitimate users with a very slow Internet connection.

Slow DoS attacks can also be generated as DDoS (distributed DoS) attacks. In this case, the attacker uses an army of hijacked computers and devices to conduct an attack on the target system at one time. Such an army of computers is called a botnet, and hijacked computers are called bots. To create a botnet, an attacker typically uses self-propagating malicious code, which scans open ports and the security flaws of computers on the Internet. If an attacker finds a computer with a security flaw, he can upload his malicious code and gain control over this computer. This computer is then part of the botnet and waits for the attacker’s order to generate an attack. The botnet will increase the volume of the attack and reduce the possibility of attacker identification because the attack is spread among a large number of computers. Accurate detection of slow DoS attacks is one of the most challenging goals in this research area [1].

The paper is divided as follows. Section 1.1 reports related papers on the topic of slow DoS attacks. Section 1.2 describes the main contribution of this paper and the proposed generator. Section 2 describes the key characteristics of slow DoS attacks. Section 3 describes the proposal of the attack generator. Section 4 describes the attack prevention capabilities of web servers and the deployment of attacks in the experimental environment against several types of the most frequently used web servers—Apache, Nginx, lighttpd, and Microsoft IIS (Internet Information Services). The used attack models and their settings are also described here. At the end of this section is a table with the results of the success of attacks against individual web servers. In Section 5, we summarize the facts about prevention and possible methods of attack mitigation. We further discuss and suggest several methods of attack detection based on the observed attacks’ behavior. The last section, Section 6, summarizes the main findings and results of this paper.

### 1.1. State of the Art

In recent years, several papers that deal with slow DoS attacks were published. Generally, these papers are focused either on the proposal and analysis of specific types of attacks, analysis of attack behavior in a particular system, methods of detection, and mitigation. Current slow DoS attacks are often very similar, but it is possible to categorize them. A possible method of categorization relates to the used version of the HTTP (Hypertext Transfer Protocol). This categorization is used, e.g., in papers [2,3], where several types of slow DoS attacks are described. However, not all currently used slow DoS attacks are described in these papers. Generators used for the experimental testing are not mentioned either.

The most current overview of application layer DoS attacks is found in [4] from 2021. The authors summarized all known facts about the characteristics and mitigation of slow DoS attacks and available tools. The current situation in the field of DDoS (distributed denial-of-service) attacks is described in [1]. In that study, their characteristics, methods of detection, and mitigation, and the current scientific challenges in this area are described and discussed. However, to be able to develop detection and mitigation techniques and to focus on newer types of techniques, a generator of these types of attacks is needed.

Some of the most well-known slow DoS attacks are Slowloris [3], Slow POST [3], and Slow Read [5]. The mentioned papers describe the main characteristics of these attacks and suggest detection methods or preventive recommendations for web servers. However, both papers did not focus on a wider area of slow DoS attacks. The study in [6] discussed the mitigation against slow DoS by monitoring the time rate and data flow of TCP connections. The work in [7] described the possibilities of increasing the web server security level by modifying its configuration, using a firewall, or modifying the network infrastructure. The study in [8] presented an intrusion prevention system (IPS) for Slowloris, Slow POST, and Slow Read attacks. The system is based on the detection of attack signatures in the HTTP and TCP content. The system is designed as a separate network filter. When an attack is mitigated, it filters the attacker’s traffic and communicates with the server to free up already occupied resources. However, the system is only suitable for detecting an undistributed attack. In the case of a distributed attack, the system does not have appropriately designed signatures and would have a long response time.

In [9], a novel advanced application-independent Slowcomm attack was presented together with the metrics for a successful attack, and test results for FTP (File Transport Protocol) and SMTP (Simple Mail Transfer Protocol) servers. However, the proposed attack model was not able to perform a continuous DoS attack; therefore, the authors discussed the possible future development of this attack, which could provide a technique for managing the continuous DoS effect. In [10], another novel application-independent attack called Slow Next was presented. The work in [11] introduced a threshold-based detection technique for this attack. However, this method is effective for Slowloris and Slow Read attacks but shows a high rate of false positives for Slow Next. In [12], the authors created and tested a method of detecting Slowloris, Slowreq, Slow Next, and Slow Read attacks based on the analysis of data on the TCP/IP transport layer. This technique monitors the progress of incoming packets at certain intervals. The advantage is a faster detection due to the absence of application data analysis and long-term communication monitoring. This method proved to be effective for the above-mentioned attacks except for Slow Next, which could not be clearly distinguished from legitimate traffic. In the case of the utilization of SDN (software-defined networks), ref. [13] proposed a machine learning framework for the detection of slow DoS attacks, and [14] deployed detection and mitigation methods against distributed Slowloris and Slow POST attacks using an SDN controller application. The study in [15] proposed a deep learning model for detecting DoS attacks in various network environments. However, all three papers did not mention the used generator for experimental testing, and thus it is hard for a reader to test his network infrastructure.

One of the biggest current challenges in the field of slow DoS attacks is the attack called SlowDrop, which was introduced in 2019 in [16]. This resource describes the attack model and tests. However, the tests are performed in an ideal environment under conditions that do not correspond to use in a real network. The source further discussed the possibilities of detection but did not provide any specific functional design for detection and mitigation. This attack can harm various protocols and server systems. Although this attack should be considered as a serious problem, at the time of writing this paper, there are still no other papers bringing new insights into the analysis, detection, and mitigation techniques of the SlowDrop attack.

In addition to these attacks, there is also another group of slow DoS attacks, focusing on the HTTP/2 protocol, which was adopted by the IETF (Internet Engineering Task Force) in May 2015 [17]. Research presented in [18,19,20,21] showed the importance of developing additional HTTP/2 security due to the discovered and tested security vulnerabilities of this protocol. This research was followed by [22,23]. The authors presented a detailed security analysis of HTTP/2 and a group of novel HTTP/2 slow DoS attack models including experimental testing against web servers. The method of detection using the chi-square test and machine learning using four different techniques was also mentioned in these papers.

In [24], a novel DoS attack called H2DoS was proposed. This attack exploits the flow control mechanism of HTTP/2. The authors also suggested preventive changes in the web server configuration. The study in [25] from 2019 presented a next-generation application DDoS called Multiplexed Asymmetric DDoS Attack, which causes the victim’s processor to overload by exploiting HTTP/2 multiplexing. HTTP/2 can also be exploited for a man-in-the-middle attack via DNS cache poisoning and a spoofed TLS (Transport Layer Security) certificate, as published in [26].

In today’s world, slow DoS attacks can infect a huge number of IoT (Internet of Things) devices. These devices have very limited computing resources and security levels. Thanks to this, they can be relatively easy to control and misused for an attack. Although these devices cannot generate large data traffic, they have more than enough resources to generate a slow DoS attack. The topic of IoT device security is thus another challenge in the field of cyber security [1]. The work in [27] described the vulnerabilities in the IoT environment using the MQTT (Message Queuing Telemetry Transport) protocol.

Many of these papers did not specify the generator used. However, a separate detailed analysis of the available DoS attack generators was provided in [28]. The authors compared and categorized a large number of available tools. Slow DoS generators were also described in an already mentioned survey [4]. All sources indicate that there is currently no tool available for Slowcomm, Slow Next, SlowDrop, and HTTP/2 attacks. During our research, we also did not find any generator for these attacks. The only slow DoS attacks addressed in this paper for which we found an available generator are Slowloris, Slow POST, and Slow Read. These attacks are contained in the slowhttptest tool, published by Sergey Shekyan in [29]. The Slowloris attack, as the most mentioned attack in the slow DoS category, is also available in various stress testing systems [30], the Pyloris tool [28], and the original Perl Slowloris script [2].

### 1.2. Contribution

This paper is a review of [31] which brings a new perspective on well-known protocol vulnerabilities and possible exploitation by adjusting the parameters of Slowloris, Slow POST, and Slow Read attacks. We implemented an attack generator, which, in comparison with other generators, brought the possibility to better adapt attacks according to the server’s security and increase the volume of the attack thanks to the simulation of the distributed form. In this paper, we expanded our previous work with more recent attacks. Our goal was to focus on the current most dangerous attacks and to create effective attack models to verify the vulnerability of network systems. We present an updated and more comprehensive attack generator, which contains a total of 11 slow DoS attacks—Slowloris, Slow POST, Slow Read, SlowDrop, Slow Next, Slowcomm, and a group of attacks focusing on the HTTP/2 protocol—Slow Read, Slow POST, Slow Preface, Slow Headers, and Slow Settings. The primary motivation for the creation of this generator was to provide a comprehensive tool for our future research, as there are no tools available to perform newer types of slow DoS attacks [28]. We especially considered the implementation of SlowDrop, Slow Next, and Slowcomm attacks into a usable generator to be the main contribution of our work. Our attack models can also be distributed and allow detailed parameter settings to obtain maximum similarity to the slow data flows of legitimate users. As a result, attacks have a higher chance of being undetected and causing maximum damage. This is the main innovation of our generator compared to other tools. By using these models, we also tested some of the most commonly used web servers. The obtained results contain novel data regarding the resistance of web servers to these attacks, specifically SlowDrop, Slow Next, and Slowcomm attacks. These results can be extremely valuable for developers and security experts to improve the security level of web servers and the detection and prevention techniques such as firewalls, intrusion detection systems (IDSs), and IPSs. Unambiguous detection of these attacks is one of the biggest challenges in the field of cyber attacks [1]. In addition, this generator can be used as a tool for further research into identifying and mitigating the impacts of these or other zero-day attacks.

Table 1 compares the selected papers from the selected slow DoS attacks’ point of view. It is clear from the table that the slow DoS attack area is increasing, and to be able to handle cyber attacks, proper security techniques and principles are needed. Further, current generators often do not allow more detailed modification of the attack’s parameters.

The research was carried out in the following steps:

- Analysis of the current state of slow DoS attacks;
- Analysis and proposal of key attack properties;
- Implementation of slow DoS attack generator;
- Experimental testing of Internet services vulnerabilities;
- Evaluation of servers’ security level;
- Prevention and detection techniques discussion and proposal.

## 2. Analysis of Slow DoS Attack Key Parameters

This section aims to analyze slow DoS attacks and find key signatures for detection. These parameters will allow the generation of authentic attacks, in order to analyze the server’s weaknesses, and to design detection mechanisms. The main key behavior of the attack is the effort to establish as many connections as possible and keep them active for as long as possible using a very slow communication. This leads to the exhaustion of all the web server’s available resources [1,32]. The attack technique differs depending on the type of attack. Attackers can simulate the slow sending of a request, limit server responses, or focus on flaws in the implementation of the protocol causing depletion of the available resources (widely used in HTTP/2 attacks) [2].

Today’s detection mechanisms monitor several parameters of the network traffic, which reach non-standard values during an ongoing attack. One of these parameters is the number of TCP connections from one IP address. An attacker has to establish a large number (hundreds or thousands) of TCP connections at once to occupy all the computing resources of a web server. However, ordinary users usually maintain only a few open TCP connections with the server when browsing web pages [6]. Another indicator of an ongoing slow DoS attack may be the duration of the established TCP connection and the rate of the transmitted data. Attackers typically send only one or a few characters in each part of an HTTP request, trying to keep the connection open for as long as possible. Therefore, it is recommended to set a threshold of acceptable connection quality to eliminate these extremely slow data flows directly to the web server. In the case of a slow legitimate user with such a slow connection, the user would not be able to download any usable web data in a reasonable time [6,33].

However, slow DoS attacks can be significantly more effective using a distributed form of attack. An attacker can launch an attack from more than one computer in the form of a single TCP connection per computer. This makes each of the attacker’s computers appear to be a legitimate user with a slow connection. The next step to reduce the probability of attack detection is to carefully set the content and timing of sending data parts between both ends. The closer the nature of the transmitted data to legitimate traffic, the more difficult accurate detection is.

### 2.1. Attacks on HTTP/x

#### 2.1.1. Slowloris

The Slowloris attack is also called Slow GET or Slow Header. The HTTP GET request is sent to the server. This request is not validly terminated due to the absence of the terminating character \r\n\r\n (double line break). The server thus waits for the next part of the request, which will contain a terminating character. This waiting is limited in the server configuration. After the timer is exceeded, the server closes the TCP connection. However, before this time expires, the attacker sends another part of the request. This keep-alive packet usually contains only a few random characters. This packet resets the timer, and then the attacker is silenced again. Then, the whole process is repeated [34]. In this way, an attacker attempts to establish as many TCP connections as possible and exhaust all free server resources that might otherwise serve other legitimate users.

#### 2.1.2. Slow POST

The Slow POST or R.U.D.Y. (R U Dead Yet) attack uses an HTTP POST request. This type of request is usually used to send data filled into Internet forms. The HTTP request header contains the Content-Length field, which specifies the size of the transmitted form of data after the request header. In this attack, the Content-Length field contains a very high value, meaning the server will expect to receive a large amount of data. The header of the spoofed request is then validly terminated, and then the request contains a small piece of data, which is usually represented by a few random characters. The attacker then waits and sends another small piece of data before the connection termination timer expires. In this way, the attacker keeps the connection active and similarly attempts to establish as many of them as possible. This leads to exhausting all available server resources [2].

#### 2.1.3. Slow Read

The Slow Read attack uses HTTP and TCP protocols. At the beginning, an attacker requests some larger data, such as an image, by sending a valid GET request to the server. An attacker sets the window-size parameter in the TCP header to a very low TCP window value. This parameter determines the amount of data that the server can send without any acknowledgment. This mechanism is used to regulate and adapt the data rate to the quality of the connection between the endpoints. The server is forced to send a response in very small parts [35]. In this way, an attacker can reach a state where the transfer of a 1 MB file can take several days. An attacker uses this technique to exhaust all available server resources.

#### 2.1.4. SlowDrop

The SlowDrop attack is one of the newest threats in the field of slow DoS attacks. This attack develops the characteristics of its predecessors, making it more destructive and less detectable. An attacker first requests the download of some content but randomly drops parts of the response from the server, simulating the dropping of packets due to poor connectivity. The server is forced to resend the dropped packets until the client’s request is finished. The attack is thus characterized by a continuous data transfer between the attacker and the victim, meaning the volume of communication over time does not contain any significant peaks [16]. Due to this feature, it is practically impossible to distinguish this attack from legitimate users based on traffic analysis. In practice, commonly used IPSs and firewalls may disconnect the legitimate user by mistake in defense, which is another possible welcome eventuality for the attacker. In addition to waste server TCP connections, SlowDrop can exhaust the victim’s network and hardware resources when requesting large amounts of data [16].

#### 2.1.5. Slowcomm

The Slowcomm attack is an application-independent attack. Using an appropriately selected invalid data content, this attack can harm various Internet services such as HTTP, FTP, or SMTP servers [9]. The principle of the attack is essentially identical to Slowloris. The attacker sends an incomplete request and the server waits for the rest of the data. During this time, the attacker attempts to generate as many such requests as possible, which leads to the occupation of all available server resources, and the server is unable to accept further requests from legitimate users. After a delay, the attacker then sends an additional piece of data, keeping the connections open. Compared to the original Slowloris model, Slowcomm includes a connection monitoring component and attempts to reconnect immediately when the server closes some of the connections. With other minor improvements, Slowcomm is much more efficient, server unavailability can theoretically last indefinitely, and it does not cause a denial of service of a victim by a sudden request flood [9]. The course of the attack can be slower without significant fluctuations in data rates, which can make detection more difficult.

#### 2.1.6. Slow Next

This attack also belongs to the category of application-independent DoS attacks. Slow Next abuses the timer between the server response and another client request within a persistent connection [10]. In this case, the attacker sends a valid request to the server, and the server responds validly. The server then waits a while to see if the attacker will request any more data. The attacker is silent for a moment and then sends another valid request. In this way, the attacker keeps a persistent connection open throughout the attack. If they manage to open more connections, they may overload the server, which will no longer be able to communicate with other users. As the communication contains valid data, the attack can easily bypass common detection systems.

### 2.2. Attacks on HTTP/2

The main benefit of the HTTP/2 protocol is the parallel processing of request streams within one connection to use network resources more efficiently and to reduce latency. HTTP/2 defines a new basic unit of communication—the frame. Each frame contains a header and data. The header consists of length, type, and flag values. A collection of frames is called a message, which forms a complete request or response. The protocol then transmits these messages bidirectionally in HTTP/2 streams. In HTTP/2, several properties are further defined to increase the efficiency of data transmission such as stream multiplexing and stream prioritization. More information can be found in [36]. On the other hand, these new features may pose new security risks. HTTP/2 connections can be memory-intensive, despite the header compression. The size of the allocated space is defined by the SETTINGS frame. This frame can be misused for a slow DoS attack by swapping, repeating individual parts of the frame, or creating new undefined parameters. An attacker can also abuse the WINDOW_UPDATE and PRIORITY frames similarly. Another option is to flood the target with a large number of small empty frames and force it to extend the processing time [22].

#### 2.2.1. Slow Read

In this attack, the SETTING_INITIAL_WINDOW_SIZE flag is set to 0, followed by a valid GET request. This tells the server that the client is currently busy; therefore, the server will wait to receive the WINDOW_UPDATE frame, which the attacker will not send. This action freezes the data stream, and the server waits until its timer expires. When the server is flooded with such connections, all free resources of the server are occupied, and communication with other users is dropped [22].

#### 2.2.2. Slow POST

For a successful attack, the attacker must set the flags of the HEADERS frame to the following values: END_STREAM: 1, END_HEADERS: 0, and send a valid POST method. This causes the server to wait for the next DATA data frame [22]. The data frame wait time depends on the server implementation. This timeout is used for a DoS attack. A larger number of such connections occupy all available server resources and cause service unavailability to legitimate users.

#### 2.2.3. Slow Preface

After a successful TCP connection is established, the attacker sends a CONNECTION PREFACE frame. This frame has the following form: "PRI * HTTP/2.0\r\n\r\nSM\r\n". After receiving this frame, the server expects confirmation of the negotiated connection parameters in the SETTINGS frame, as well as the valid request itself. However, the attacker never sends this request at all. This forces the server to wait until the server closes the connection itself [22]. This timeout is sufficient to cause the denial of service for legitimate users.

#### 2.2.4. Slow Headers

This attack can be performed using a GET or POST request. The attacker, similar to the HTTP/2 Slow POST attack, uses the same HEADERS flags of the frame. If the POST request is used, the attacker sends a HEADERS frame with the END_HEADERS: 0 and END_STREAM: 0 flags. In the case of a GET request, the attacker sends END_HEADERS: 0 and END_STREAM: 1. The END_HEADERS: 0 flag says that the HEADERS frame is not complete and must be followed by another CONTINUATION frame. In contrast, END_STREAM: 1 indicates the end of a valid HTTP/2 stream. The server will wait for the CONTINUATION frame with more data, but the attacker will never send it. Then, the server closes the connection [22]. This timeout is abused to cause the denial of service for legitimate users.

#### 2.2.5. Slow Settings

The attacker sends a valid GET or POST request, including the SETTINGS frame. The SETTINGS frame must be confirmed by the SETTINGS: 0 frame [17]. The server responds by confirming the client’s SETTINGS and sends its SETTINGS frame, which the attacker no longer confirms. The server will wait and close the connection with a SETTINGS_TIMEOUT error [22]. This timeout is abused to cause the DoS for legitimate users.

### 2.3. Summary of Selected Slow DoS Attacks

Table 2 summarizes the selected slow DoS attacks presented in this paper and mentions the main signs (main principles) of the attacks. These attacks are divided into two groups (HTTP/x, HTTP/2) as in this paper. Most attacks attempt to behave (or directly cause this condition) like users with a slow Internet connection. Therefore, their detection is not as easy as in the case of volumetric DoS attacks.

## 3. Proposal and Implementation of Attack Generator

We propose a generator of all the slow DoS attacks mentioned in Table 2. The primary motivation for creating the proposed slow DoS attack generator was the absence of freely available attack generators. The proposed attack generator is implemented as a Linux console application written in the Python programming language. The generator requires a Linux operating system with iptables and a Python interpreter 3.8.x or higher. Other dependencies are the following Python libraries. Generating the required amount of traffic and parallelizing processes is achieved by the *requests* and *threading* libraries. Moreover, the *sockets*, *hyper-h2*, *hyperframe*, *hpack*, *pandas*, and *plotly* libraries are also used.

The attack is started by a specific command in the terminal. The command must contain the type of attack and the target of the attack, which are mandatory parameters for all attacks. Other parameters vary depending on the selected attack. The user can use the program’s help to see all available settings. A more detailed description of the parameters is provided for all test scenarios in Section 4. As soon as a valid command is executed, the generator starts generating an attack. The generator periodically sends predefined data to the server according to the set parameters. In the case of some attacks, the number of established connections is monitored. When it drops, the connections are re-established. The attack can be terminated by pressing the Ctrl + C keys. The established connections with the victim remain for some time, and it is possible to observe their gradual termination according to the set timeouts of the server.

**Figure 1:**
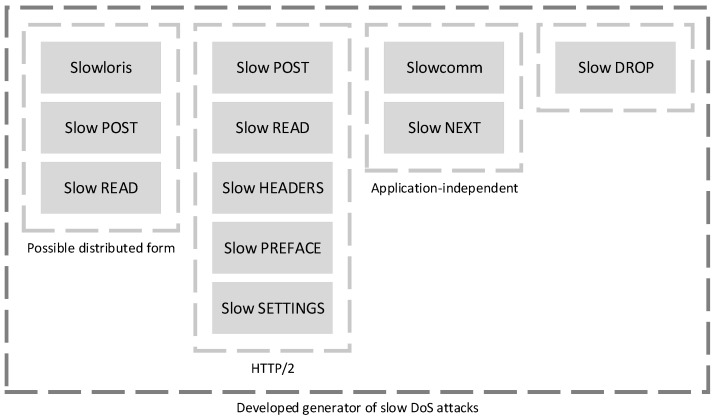
Individual parts of implemented slow DoS generator.

The generator core is divided into several parts (scripts) according to the attack type. Figure 1 shows the individual parts from which the proposed generator is implemented.

The first part includes Slowloris, Slow POST, and Slow Read. Our generator allows us to set the attack’s parameters more precisely than in competing tools and also launch an attack in a distributed form. We believe that by more precisely adapting the attack to the server configuration, it is possible to achieve a successful attack even with applied preventive protection. Many detailed attack parameters can be specified in the generator. General input parameters include the output network interface, destination IP address, destination website URL, starting port, port increment step, total number of connections, time between individual parts of data, delay between individual clients, and setting the number and IP addresses of bots in case of a distributed attack. Other input parameters depend on the selected type of attack. For example, it is possible to specify the initial HTTP request’s content, the size of the randomly generated data, the size of the TCP window, and the path to the requested file.

A distributed attack simulation is currently only available for the following three attacks: Slowloris, Slow POST, and Slow Read. In this scenario, the virtual bots will be simulated on the local subnet to which the generator is connected. The subnet should have enough address space. In our experimental environment, we used a subnet with a netmask 255.0.0.0, which provides an address space for approximately 16 million computers. Virtual bots are simulated using the Address Resolution Protocol (ARP) data spoofing. In the first step, the generator receives from the user a range of IP addresses on which to simulate the bots. Then, the generator sends an Internet Control Message Protocol (ICMP) *Echo request* to these addresses. This will cause other devices on the subnet to query the MAC addresses for these IP addresses using ARP. Subsequently, the generator sends out modified ARP messages, in which it spoofs its own MAC address. This will ensure that traffic routing on bot IP addresses is actually directed to the generator. Using this technique, it is possible to imitate a situation where an attack is coming to the web server from many different Internet addresses. However, the limitation is that the bot subnet must not be hidden behind Network Address Translation (NAT) in the test environment.

The next part of the generator includes HTTP/2 attacks—Slow POST, Slow Read, Slow Headers, Slow Preface, and Slow Settings attacks. It accepts the following input parameters: victim’s IP address and port, attack type, and the number of connections. The attack model also contains a component to verify the availability of the target server.

The application-independent Slowcomm and Slow Next attacks are implemented in the next generator part. The input parameters are the IP address and port of the victim, the type of attack, and the number of connections, and, optionally, the content of data sent by the attacker, two types of timeouts (to make the attack more effective depending on the server configuration), and the number of processor threads. The ability to set any port and data payload allows the use of attacks to exploit various protocols.

The last part of the generator is focused on the SlowDrop attack. Depending on the needs of the scenario, the attack can be modified with input parameters: the number of threads generating requests, the time interval between threads to spread the attack, the interval for sending a new request, and the incoming packet drop ratio. Optionally, it is possible to modify the content of the HTTP request due to the higher server load. It is recommended to modify the *user-agent* for hiding the Python client, *cache-control* for non-caching, and *accept-encoding* to increase fragmentation, and to set *connection: keep-alive* to prolong the TCP connection (if the server supports persistent connections). The first step to launch a SlowDrop attack is an HTTP GET request for a larger amount of data. Subsequent answers need to be dropped with some degree of randomness. The native Linux iptables firewall was chosen for this purpose. The iptables program contains the ipt_random and ipt_statistic modules used primarily for the load balancing function. However, this function can be used to determine the percentage of forwarded or dropped packets, which is very suitable for SlowDrop attacks. On the contrary, the authors of the attack in [16] for implementation chose the NFQUEUE tool and cited [37] dealing with iptables acceleration using NFQUEUE with GPU parallelization. According to this paper, the decision speed in parallel processes of the network rules is up to 43 times higher than on a regular computer with iptables. We used the python-nfqueue library to implement this solution for our generator. However, the resulting solution was not as efficient and fast as using iptables directly in the Linux kernel. The NFQUEUE queue was overflowing, and more packets were inadvertently dropped than required. Our generator does not assume parallelization on the GPU; therefore, we decided to use iptables for this purpose. The main advantage is that iptables drop packets at lower layers and are not forwarded to the NFQUEUE queue through the application layer. Therefore, the application daemon is no longer burdened by these packets. This feature thus better captures the slow DoS attacks, attempting to minimize the load on the attacker’s machine.

### Tools Comparison

To evaluate the proposed generator in terms of the variability of individual parameters, we selected one of the most used tools for Slowloris, Slow READ, and Slow POST attacks. We compared the proposed generator with the slowhttptest [38], Slowloris [39], PyLoris [40], and R.U.D.Y. [41] tools.

Table 3 compares the proposed generator and the slowhttptest, Slowloris, and PyLoris tools from the editable parameters’ point of view. The table is divided into two main parts: the first part is comparing attack-specific parameters, and the second part concerns general parameters. If the tool allows editing of a parameter, the **✓** symbol is used (also the preset values are shown); in the other case, the **✗** symbol is used. Due to a fully editable Slowloris header, it is possible to generate specific values. All preset Slowloris header parameters are shown in Listing 1. Table 4 compares the proposed generator with the slowhttptest tool that is able to generate the Slow READ attack.

Listing 1 **Default parameters of Slowloris header used by the proposed generator.**

GET /?654865241562456 HTTP/1.1

Host:

User-Agent: Mozilla/4.0 (compatible; MSIE 7.0; Windows NT 5.1;

Trident/4.0; .NET CLR 1.1.4322; .NET NET CLR 2.0.50313;

.NET CLR 3.0.4506.2152; .NET CLR 3.5.30729; MSOffice 12)

Content-Length: 42

Table 5 compares the proposed generator and the slowhttptest and R.U.D.Y. tools. These tools also enable the generation of the Slow POST attack. The advantage of the R.U.D.Y. tool is the implementation of Tor usage. Unlike other tools, our tool allows setting the starting port and the step that is used when the port is changed. As in the Slowloris attack, the proposed tool enables a fully editable Slow POST header. The preset Slow POST header is shown in Listing 2.

The comparison showed that the proposed generator is the only one capable of the bot simulation. Using this parameter, it is possible to multiply the impact of the generated attack. Moreover, the proposed generator provides a fully editable header used in the attack, and a port step function. This function periodically changes a port within the attack.

Listing 2 **Default parameters of slow POST header used by the proposed generator.**

POST /textform.php HTTP/1.1

Host:

User-Agent: Mozilla/4.0 (compatible; MSIE 7.0; Windows NT 5.1;

Trident/4.0; .NET CLR 1.1.4322; .NET NET CLR 2.0.50313;

.NET CLR 3.0.4506.2152; .NET CLR 3.5.30729; MSOffice 12)

## 4. Experimental Verification of the Generator

**Figure 2:**
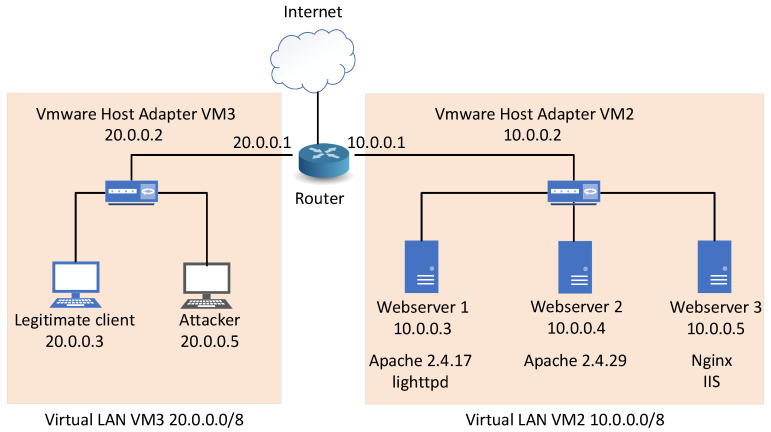
Experimental network topology.

This section describes an experimental environment which was created to verify the functionality of the proposed generator. The environment was created by virtualization using the VMware software. The parameters of the virtual machines are specified in Table 6. The topology of the environment is shown in Figure 2. It contains a server subnet that can be easily extended by other types of servers. For experimental purposes, three servers are used, each with a different type of used web server. There is also a client subnet that contains user and attacker computers. This subnet has a 255.0.0.0 netmask providing enough address space to simulate distributed DoS attacks. In all scenarios, only one client is used, meaning the server has a minimal load and almost ideal conditions to handle the attack. The router and VMware host adapters are the backbone network devices on which detection and mitigation software can be developed and tested. The entire environment is hidden behind NAT and connected to the Internet via the router, due to the installation of the necessary software and updates.

In total, vulnerabilities were tested on five web servers—2x Apache2, Nginx, lighttpd, and Microsoft IIS. We chose these web servers because they are among the most used on the Internet. We primarily used the latest server versions at the time of the test. When choosing Apache versions, we also considered usage statistics in [42] to make our results relevant to as many users as possible.

Except for the lighttpd server, these servers already have DoS protection pre-installed. Therefore, servers should be protected from various threats. In the case of slow DoS attack resistance, the tolerance level of slow connections set by web server developers is crucial. A significant percentage of web server administrators do not pay enough attention to additional server configurations, prevention, and deployment of additional protection systems [1]. Therefore, in these test scenarios, the web servers were left in the default configurations, and their ability to resist the selected slow DoS attacks without significant configuration changes was monitored. This is to reflect the common situation where server administrators often do not pay extra attention to configuration modifications and leave the server in an out-of-the-box configuration. Only pre-installed attack protection is enabled on the servers. Details are described in the following subsections.

The tests were performed as follows. A legitimate client periodically sent a request to the server to view a web page every second. Each request was initiated by a TCP handshake within a new connection. In this way, the client verified the availability of the server and whether a DoS condition occurred. Once an attacker launched an attack on a server, the client monitored server response failures and delays.

The courses of the test scenarios are shown in graphs, where the X-axis represents the time. Most tests are displayed at a scale of 30 seconds to make the server behavior and connection changes clearly observable. There were no further major changes in the longer time intervals. In some scenarios, the server responds to the attack with a longer delay. This is based on the configured server timeouts. Therefore, some graphs display events at longer intervals. The main effort is to make the changes and behavior of the server in the graphs clearly visible. The left Y-axis represents the number of TCP connections, and the right Y-axis represents the web server availability by the percentage of legitimate user processed requests. The green curve represents established TCP connections, the orange curve represents pending TCP connections, the red curve represents closed TCP connections, and the blue curve represents the success rate of legitimate user requests.

### 4.1. Apache Web Server

The primary tested web server was Apache 2.4.29, which, by comparison in [42], is the second most used version of Apache 2.4 (on 29 March 2021). This server already has a pre-installed security module against DoS attacks. The main parameters of the server configuration are shown in Listing 3.

Listing 3 **Configuration file of Apache 2.4.29.**

Timeout         300

KeepAlive        On

MaxKeepAliveRequests   100

KeepAliveTimeout∼5

 

<IfModule reqtimeoutmodule>

  RequestReadTimeout header = 20-40,MinRate = 500

            body = 10,MinRate = 500

</IfModule>

 

<IfModule mpmpreforkmodule>

  StartServers      5

  MinSpareServers    5

  MaxSpareServers    10

  MaxRequestWorkers   150

  MaxConnectionsPerChild 0

</IfModule>

Parameters in the mpm_prefork module, such as MaxRequestWorkers, specify the maximum number of clients that can be served simultaneously. It is important to choose this number carefully to avoid CPU and RAM overload. The ideal number can be estimated based on the average size of the httpd process and the server load of other processes. In the case of our server with 2 GB of RAM, we kept the default value of 150.

The value RequestReadTimeout is a timer that determines how long the server waits to receive the request or part of it. Another important parameter is KeepAliveTimeout, which specifies the time during which the client must send a complete request. Otherwise, the connection will be terminated by the server. Other related parameters such as LimitRequestBody, LimitRequestFields, LimitRequestFieldsSize, LimitRequestLine, and LimitXMLRequestBody are used to limit other types of attacks. However, an attacker can estimate these parameters by thorough testing and adapt the attack by carefully setting the attack parameters.

The secondary Apache server was version 2.4.17. Despite its advanced age and low worldwide usage [42], this server was used only for verifying the functionality of the attack, as the level of security against slow DoS is low. The server was left in the default configuration. All attacks against this server were successful.

#### 4.1.1. Slowloris

The Slowloris attack was set as follows: generating 500 TCP connections, interval 2 seconds between parts of the HTTP request, interval 10 milliseconds between individual TCP connections. This setting should create enough traffic to congest the server. The data sent by the implemented attack model are shown in Listing 4. The first packet contained the request type, HTTP protocol version, and the parameters Host, User-Agent, and Content-Length. The keep-alive packets contained the string X-a: b.

Listing 4 **HTTP content of Slowloris attack model.**

GET /?89018286261135 HTTP/1.1

Host: 10.0.0.2

User-Agent: Mozilla/4.0...

Content-Length: 42

X-a: b

X-a: b

...

**Figure 3:**
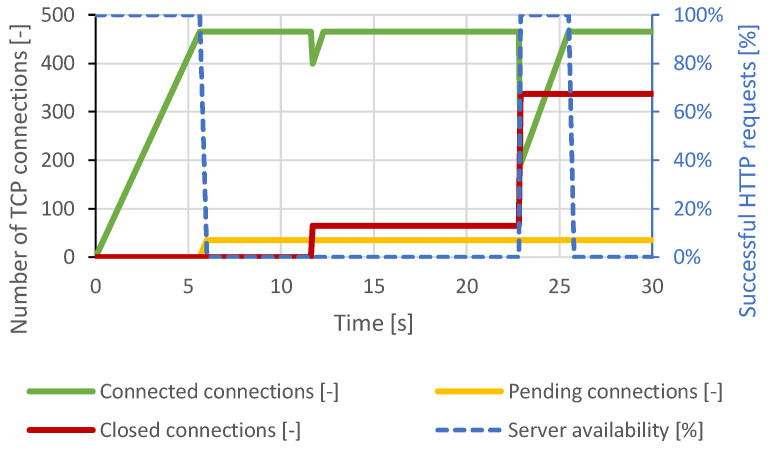
Slowloris attack against Apache server.

Immediately after connecting approximately 460 TCP connections, all server resources were exhausted, and other TCP connections remained pending. Therefore, a DoS state had occurred. A closing of several TCP connections had occurred around the 12th second of the test, but the attacker reconnected all the impacted connections. Around the 22nd second, the intervention of the DoS prevention module is visible. The server canceled the initial TCP connections, which were open too long. At this point, the server was available again, but within seconds, the generator re-established the lost connection and exhausted all server resources again. The course of this attack is shown in Figure 3. In the case of a distributed attack, the results were identical.

#### 4.1.2. Slow POST

In the Slow POST attack model, the Content-Length was set to 1,000,000 bits. Due to the low upload speed, the server would wait for this portion of data for several days, which is sufficient for this attack. The other parameters were the same as in the Slowloris attack scenario. A preview of the data sent by the implemented attack model is shown in Listing 5. The first packet contained only the request type, the HTTP protocol version, the parameters Host, User-Agent, and Content-Length, and then the first part of the data. As the keep-alive packet, the attacker repeatedly sends a randomly generated character representing the data from the Internet form.

Listing 5 **HTTP content of Slow POST attack model.**

POST /textform.php HTTP/1.1

Host: 10.0.0.2

User-Agent: Mozilla/4.0...

Content-Length: 1000000

 

name=zzzzzzzzzzzzvvvvvvvvveeeeeeejjjjjjjjccc...

**Figure 4:**
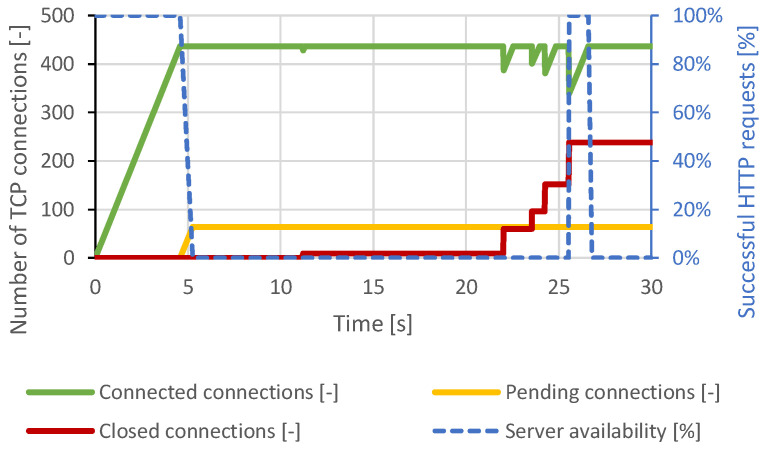
Slow POST attack against Apache server.

The results were similar to the Slowloris attack. After reaching the maximum service capacity of the server, the attacker’s connections remained open, as the attacker sent the data at a sufficient frequency. The DoS state was reached again. As the Slowloris attack, the server began terminating the initial connections. These connections were re-established by the attacker. The course of this attack is shown in Figure 4. The course of the distributed attack was very similar, but the server attempted to close the connections continuously after the 20th second of the test.

#### 4.1.3. Slow Read

**Figure 5:**
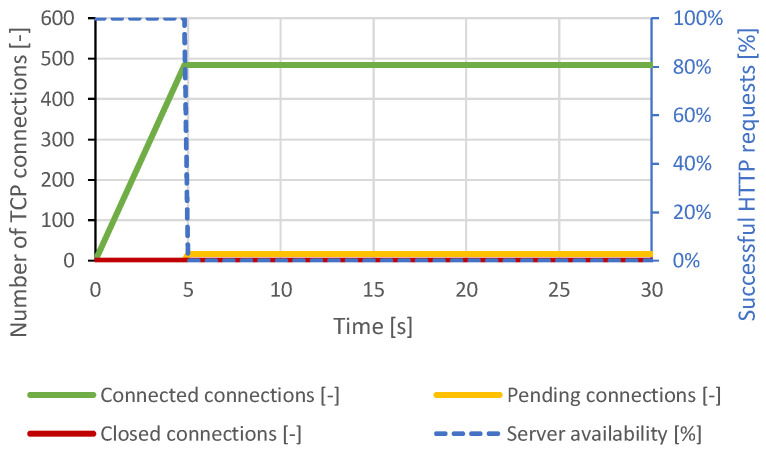
Slow Read attack against Apache server.

During the Slow Read attack, the generator was set as follows: 500 TCP connections and window size 10 bits. From previous tests, it can be seen that the upper limit of currently open connections on the Apache server is in the range of 400–500 connections. The attacker requested a 1 MB jpeg file representing a usual image on a web page. From the graph in Figure 5, it is evident that the server managed to handle less than 500 connections, and the DoS state lasted for the rest of the test scenario without any fluctuations. For the distributed Slow Read attack, the results were identical.

#### 4.1.4. Slowcomm

**Figure 6:**
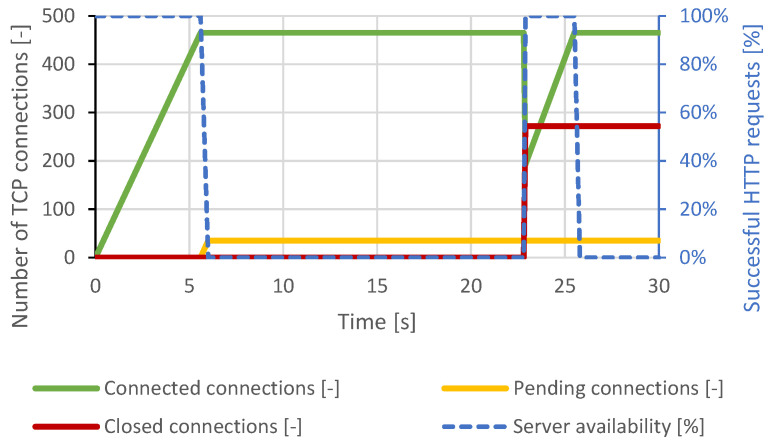
Slowcomm attack against Apache server.

The Slowcomm attack had almost identical results to the Slowloris attack, which was expected due to the same principle of attack. Our Slowloris generator has a built-in function for restoring closed connections, meaning it is practically not different from a Slowcomm attack. The course of this attack is shown in Figure 6. The difference is that Slowloris focuses only on the web service, while the Slowcomm attack generator was developed to attack various application protocols. We intend to investigate the impact of this attack on other services in future work.

#### 4.1.5. Slow Next

**Figure 7:**
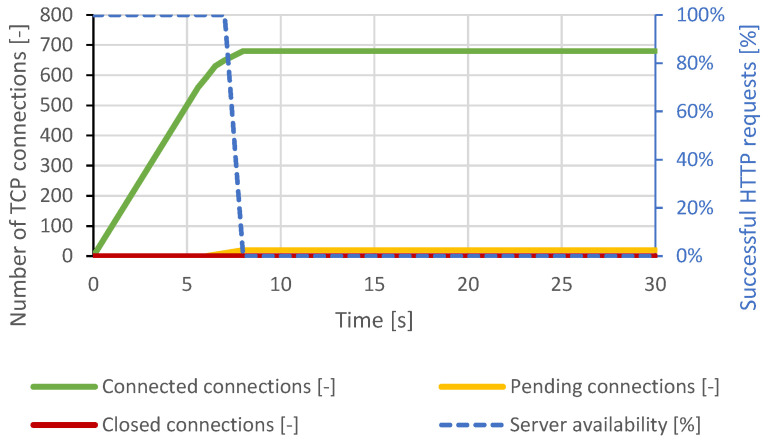
Slow Next attack against Apache server.

In this scenario, it was necessary to increase the number of connections because the server was stressed differently than in previous attacks. It proved sufficient to generate 700 concurrent connections by sending additional bits of data every 4 seconds to invoke the DoS state. Once the attacker established approximately 680 connections, the server could no longer serve other users. As it can be seen from Figure 7, this condition lasted throughout the attack.

#### 4.1.6. SlowDrop

**Figure 8:**
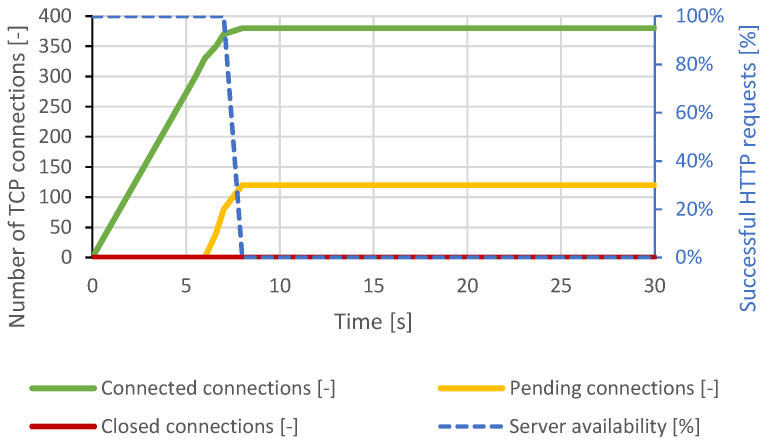
SlowDrop attack against Apache server.

The SlowDrop attack scenario was based on previous test scenarios and attempted to open a 500 TCP connection and request an image download of approximately 500 kB representing a usual web image. The drop ratio was set at 60%. This packet drop ratio proved to be optimal, as the transmission of the entire image was extended to several tens of seconds. This time is sufficient to reach the DoS condition and also not to terminate the transfer by the server. The course of this attack is shown in Figure 8. The Apache server could only handle approximately 380 connections. Then, the server exhausted all available resources and did not respond to new connection requests from the legitimate user. This condition lasted continuously until the end of the attack.

#### 4.1.7. HTTP/2 Attacks

**Figure 9:**
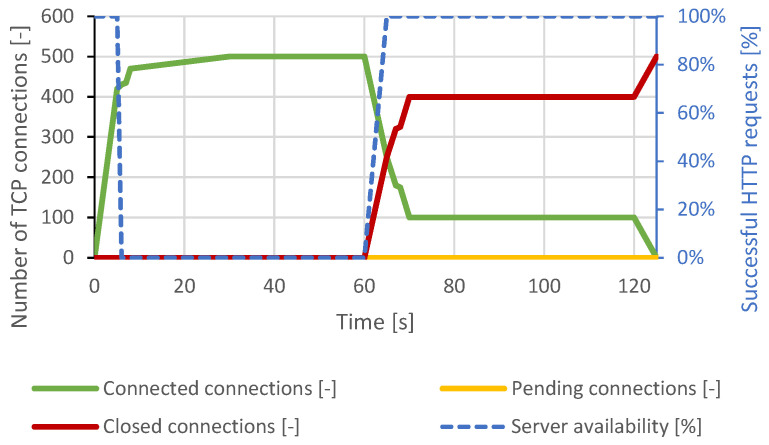
Slow Preface attack against Apache server.

Implemented attacks on the HTTP/2 protocol were able to establish and maintain a connection with the server only in the case of Apache 2.4.17. Only the Slow Preface attack was successful in the attack on Apache 2.4.29, which is shown in Figure 9. The attack established approximately 500 connections. Due to the configuration of the server and its timers, the server closed all the attacker’s connections after 60th second. During this time, however, a denial of service to the legitimate user was achieved.

### 4.2. Nginx

Another tested server was Nginx 1.14.0. This server already has a pre-installed module for slowing down DoS attacks, but other modules that help distribute the load only appear in the paid version of the server. In the free version used in this test, the security module contains parameters similar to the Apache server, e.g., client_header_timeout and client_body_timeout, in order to terminate the slow HTTP data streams. The default value for both parameters is 5 seconds. Another crucial parameter is limit_req_zone, which limits the number of HTTP requests per client. The default value is set to 30 requests per minute or one request per 2 seconds. The last important parameter is limit_conn_zone, which limits the number of TCP connections from one IP address. This protection should protect the web server from slow DoS attacks coming from one station. However, this protection should be ineffective for distributed attacks.

#### 4.2.1. Slow Read

**Figure 10:**
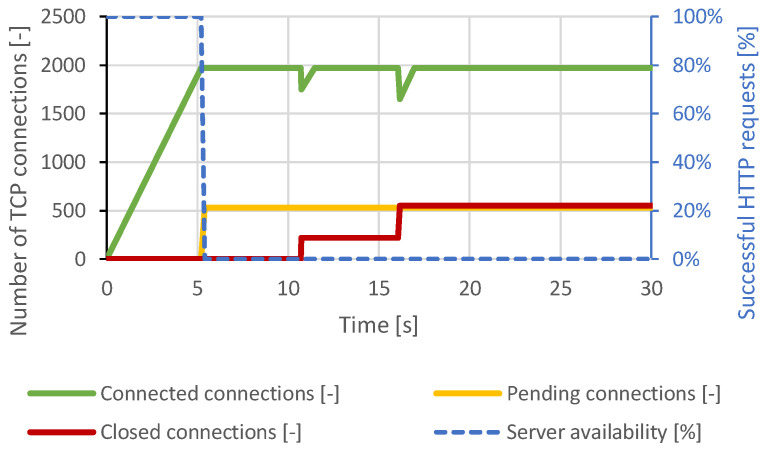
Slow Read attack against Nginx server.

In the case of the Slow Read attack, the generator settings used were the same as those for the Apache server attacks, but the total number of TCP connections was increased to 2500 due to the higher expected server performance. Approximately 1900 connections were established, and then all the server resources were exhausted. As it can be seen in Figure 10, the server attempted to terminate some connections. However, the generator restored these connections. The DoS state lasted throughout the attack. Distributed attacks had very similar results.

#### 4.2.2. Slowcomm

**Figure 11:**
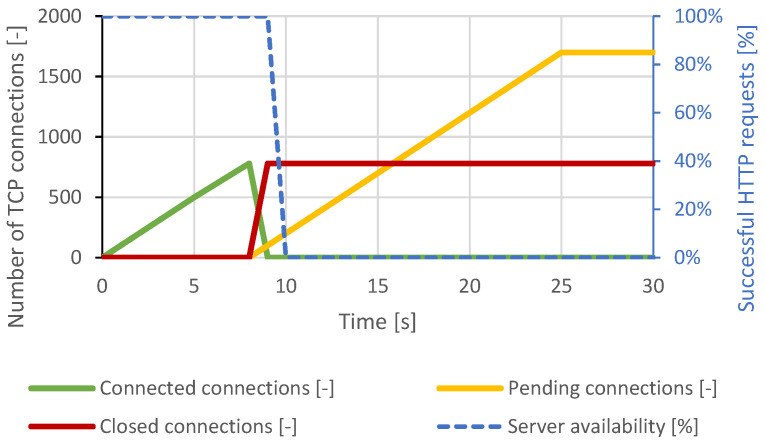
Slowcomm attack against Nginx server.

Nginx was much more resilient than Apache due to its architecture. Based on the results of the previous attack, this scenario was set to establish 2500 connections and then send data every 2 seconds. However, when approximately 800 connections were established, the server’s security mechanisms began terminating the connections, and the attack was mitigated. Paradoxically, this led to a server overload and communication interruption with the legitimate user. The course of the attack is shown in Figure 11.

#### 4.2.3. SlowDrop

**Figure 12:**
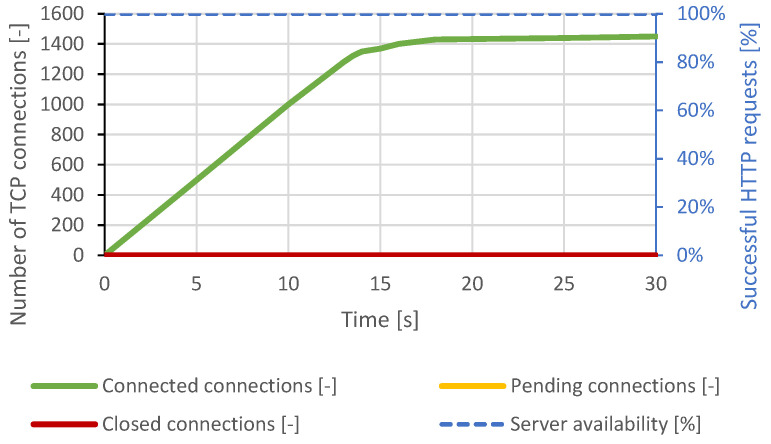
SlowDrop attack against Nginx server.

The SlowDrop attack failed to cause a service failure on the Nginx server. The generator was able to produce and maintain approximately 1400 TCP connections, but the server was able to process all of the attacker’s connections. The legitimate client was served without a noticeable delay. The course of the attack is shown in Figure 12.

#### 4.2.4. HTTP/2 Attacks

The Nginx server is immune to all implemented HTTP/2 attacks. An attacker can establish TCP connections, but they are closed by the web server.

#### 4.2.5. Other Attacks

Slowloris, Slow POST, and Slow Next attacks were ineffective against this web server due to the event-driven architecture of the web server. All attacker invalid connections were closed shortly. The web server responded to legitimate users during the attacks without any delay, meaning DoS attacks were unsuccessful. Distributed attacks had the same results.

### 4.3. Lighttpd

The next tested web server was lighttpd 1.4.55. This lightweight web server does not contain any advanced settings and elements for protection against slow DoS attacks.

#### 4.3.1. Slowloris

**Figure 13:**
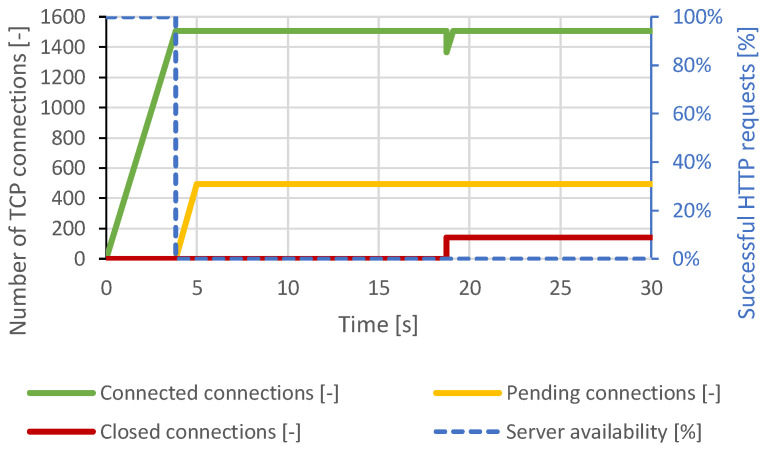
Slowloris attack against lighttpd server.

The lighttpd server is optimized to handle a large number of requests at once. The Slowloris attack scenario used the same attack generator settings as the Apache server attack scenario, but the total number of TCP connections was increased to 2000. This value was chosen based on several previous attempts to be able to cause a DoS condition. In this test scenario, lighttpd could handle approximately 1500 connections during an attack. Once the server reached this maximum, a DoS effect occurred and lasted throughout the test. The distributed Slowloris scenario had the same results. The course of the attack is shown in Figure 13.

#### 4.3.2. Slow POST

**Figure 14:**
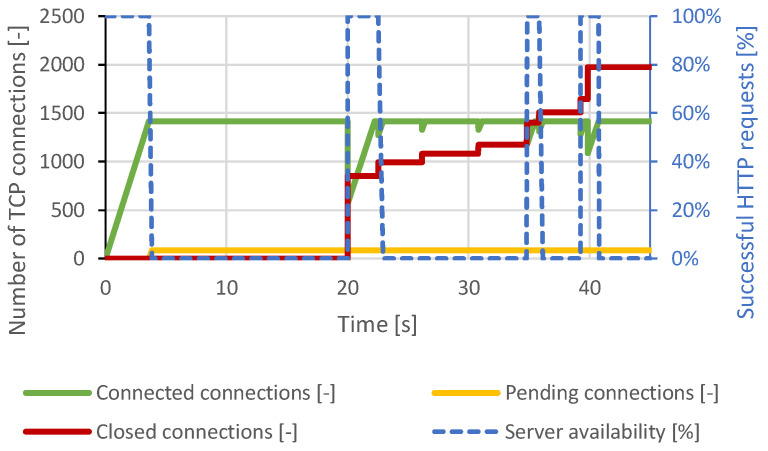
Slow POST attack against lighttpd server.

The Slow POST attack scenario used the same generator settings as the Apache server attack, but the total number of connections was increased to 1500. This value was chosen based on several previous attempts to be able to cause a DoS condition. From the course of the attack in Figure 14, the server attempted to terminate some of the connections. However, all closed connections were restored by the generator. Once all free server resources were occupied, the server became unavailable to legitimate users. The distributed attack had very similar results, but there were no such frequent fluctuations and connection termination.

#### 4.3.3. Slow Read

**Figure 15:**
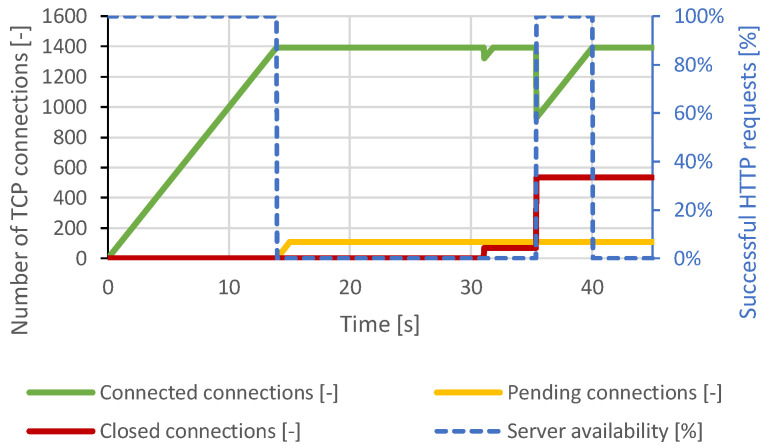
Slow Read attack against lighttpd server.

In the case of the Slow Read attack, the same generator settings as in the scenario with the Apache server were sufficient to cause a DoS condition. The course of the attack is visualized in Figure 15. An attempt to terminate a large number of TCP connections can be observed around the 20th second after all available server resources were exhausted. However, the TCP connections were re-established, and the DoS state was reached again. The distributed attack produced very similar results.

#### 4.3.4. Slowcomm and Slow Next

**Figure 16:**
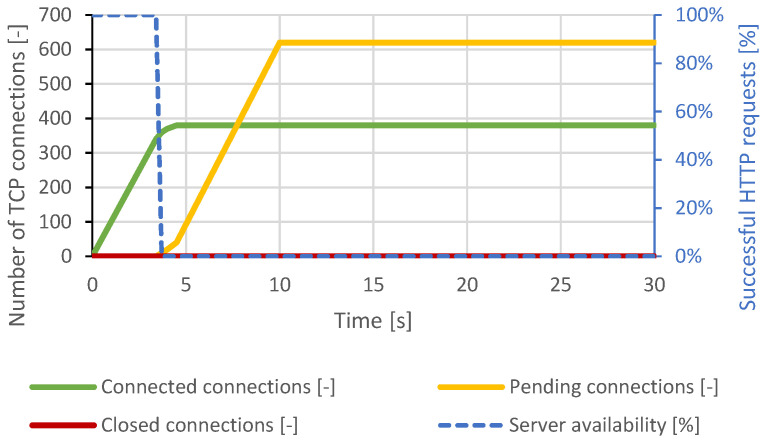
Slowcomm attack against lighttpd server.

Due to the low resource requirements of the server, the number of connections of both attacks was set to 1000 to increase the server load and exhaust the server’s resources. This number was chosen based on previous attempts to achieve a DoS condition. After establishing almost 400 connections, the service was denied to a legitimate user. This condition lasted throughout the attack. The course of the attack is shown in Figure 16.

#### 4.3.5. SlowDrop

**Figure 17:**
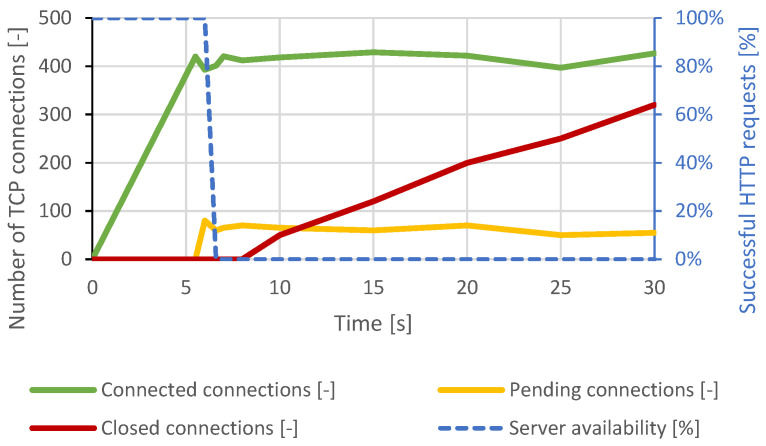
SlowDrop attack against lighttpd server.

The SlowDrop scenario had the same settings as the Apache attack scenario. The attacker opened 500 connections and requested an image of 500 kB, and a drop rate of 60%. The server handled approximately 400 concurrent connections. Other connections, including the legitimate user, were denied. The course of the attack is shown in Figure 17.

#### 4.3.6. HTTP/2 Attacks

These attacks could not be performed due to the missing HTTP/2 support in lighttpd 1.4.55. The server supports this protocol from version 1.4.56 and above [43], which was not yet released at the time of this testing.

### 4.4. Microsoft IIS

The IIS server configuration offers several parameters that can be used to secure server vulnerabilities to slow DoS attacks [7]. The maxAllowedContentLength, maxQueryString, and maxUrl parameters in the <RequestLimits> element are used to limit the attributes of the HTTP request. In the <headerLimits> element, admin can adjust the size of the HTTP header that the web server accepts. We left these values at the default state. The values in the <limits> and <WebLimits> elements directly affect the server’s slow connection behavior. We left the default values again, i.e., connectionTimeout = 00:02:00, headerWaitTimeout = 00:00:00, and minBytesPerSecond = 240.

#### 4.4.1. SlowDrop

**Figure 18:**
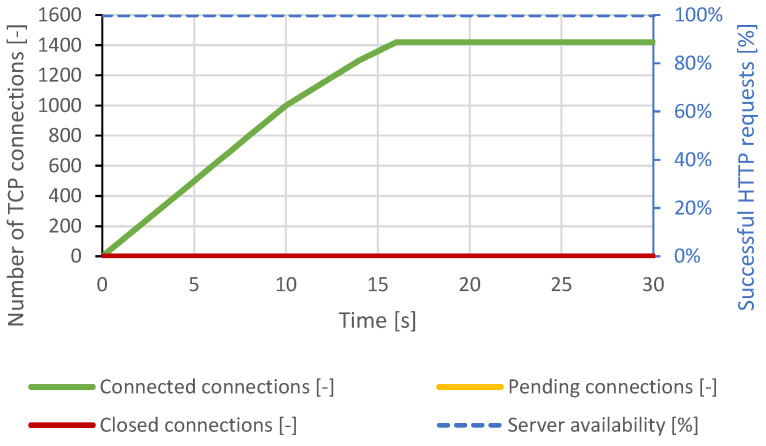
SlowDrop attack against IIS server.

After several attempts, our virtual machine with an attack generator managed to produce a maximum of 1400 connections, which was not enough to cause the DoS effect. IIS was able to handle all connections. Possible optimization of the SlowDrop attack could produce better results. The course of the attack is visualized in Figure 18.

#### 4.4.2. HTTP/2 Attacks

Microsoft IIS 10.0 supports HTTP/2 for encrypted communication only. The *h2c* (hypertext-to-cleartext) protocol is implemented over the TCP protocol, while *h2* works on the TLS protocol. Therefore, the implemented slow DoS attacks on HTTP/2 will not work on IIS 10.0, as our attack generator does not yet support encrypted communication.

#### 4.4.3. Other Attacks

None of the tested attacks could cause server failure and denial of service to a legitimate client. IIS was able to process all of the attacker’s connections that the attack generators were able to produce.

### 4.5. Summary of the Tests

The results of testing all types of investigated attacks against the mentioned servers are summarized in Table 7. An attack was considered successful if a legitimate client noticed a continuous service outage for more than 6 seconds. We determined this value based on the estimated user experience. The user would probably notice a smaller delay and, exceptionally, tolerate it. With a longer delay, it is very likely that the user would close the website. This successful invocation of the DoS state in a given scenario is indicated in the table by the symbol **✓**. Otherwise, when there was no such long service outage, there could only be a delay in response, but the service was responsive. In this case, the attack was considered unsuccessful and is marked with the symbol **✗**.

## 5. Discussion on Detection and Mitigation

The testing of web servers in the previous section proved the effectiveness of the generator and its suitability for analyzing the security of web servers. The results show that for some servers, security against the investigated attacks is sufficient already in the out-of-the-box state. Other servers need to be additionally secured either with a more careful configuration or other network protections.

The basic and simplest prevention against slow DoS attacks is to keep the computer system up to date. Another option is to use security modules and third-party systems. However, this approach is not always sufficient. In some cases, it only allows reducing the strength of the attack. Another method is the use of specialized intrusion detection and prevention systems. In addition, there are a few general steps to increase the web server’s security and prevent slow DoS attacks [1,8]:

- Limit the time of open connections with each IP address to what is necessary;
- Limit the maximum number of open connections per user and IP address;
- Limit the waiting time of the server to receive and send a request, and after this time, close the connection;
- Use the maximum computing capacity of the server to serve as many users as possible;
- Create HONEYPOT servers within the network;
- Use multiple servers with load balancing.

The simplest prevention can be created by properly configuring the web server and the firewall [33]. The settings should then be adapted to the particular server according to its content. For example, the server should be stricter in terms of attacker connections and data transfer times for a simple website with a small data content than for more complex web applications with large downloadable data. The disadvantage of restricting access from a single IP address may be the fact that an attacker can be hidden in the local network behind NAT. Thus, a countermeasure could restrict other legitimate users in the subnet. Mitigation should always focus on specific suspicious connections defined by the source IP address and port. Furthermore, limiting the connection time should take into account different situations, as communication in the Internet environment can be heterogeneous.

Slowloris, Slow POST, and Slowcomm attacks can be easily traced according to specifically modified data requests. Detection should have a certain tolerance set to distinguish between the attacker and a legitimate user. Only when the number of such connections is exceeded and it could disrupt the stability of the server should the mitigation technique block the connection and release the server’s occupied resources. Thus, it is possible to use specialized tools for the detection and mitigation of individual attacks, such as the SDToW tool [44]. This tool is for the detection and mitigation of Slowloris attacks in wireless mesh networks using a device called a concentrator. Antibot and antispam tools that limit the reception of GET and POST requests can also be useful [45]. They can help mitigate the beginning and effect of an attack. However, these antispam tools are used by web programmers rather than server administrators.

Slow Read attacks for all HTTP versions can be detected based on the extremely small size of the TCP window. However, for unambiguous detection, it is necessary to also monitor other parameters such as the speed of transmitted data, the number of these slow connections, and the specification of the required data. Here, it is necessary to carefully choose the detection threshold and determine which flows can still be tolerated and considered legitimate, and which should be terminated.

The Slow Next attack is much more difficult to detect due to the legitimate communication. For detection, it is necessary to monitor all connections and monitor the delay between individual requests. The maximum limit of waiting for the next request within the connection should be defined, and with a higher number of such connections, this limit should be reduced. This can prevent server congestion. The detection mechanism should look for similarities between request delays and recurring requests. These connections then indicate a Slow Next attack and can be terminated.

There is currently no reliable detection technique for the SlowDrop attack. The attack mimics the behavior of a legitimate user too accurately. Theoretically, it could be possible to use neural networks to detect attacks as an anomaly. An attacker is characterized by data retransmission. Setting an acceptable retransmission rate based on the amount of data could help to prevent this attack.

Attacks on the HTTP/2 protocol can be detected by the occurrence of signatures described in Section 2.2. Some of these attacks involve sending non-standard HTTP/2 frame parameters, meaning the detection should be accurate. For attacks that simulate a legitimate slow user, such as Slow Read, the detection mechanism should specify a tolerance of the data rate and the number of connections for accurate attack detection, similar to Slow Read for HTTP/1.1.

## 6. Conclusions

The test scenarios proved the functionality of the created slow DoS attack generator, in which the DoS state was successfully invoked on most web servers. However, Microsoft IIS proved to be the most resistant to these attacks. It was able to overcome all attacks and maintain the quality of service for users in all tested scenarios. Nginx 1.14.0 showed resistance to most attacks. Thanks to its architecture, it passed all attacks without denial of service, except for the Slow Read and Slowcomm attacks. A surprising result, however, is the vulnerability of the Apache 2.4.29 web server, which is still vulnerable to Slowloris, Slow POST, Slow Read, Slowcomm, and Slow Next, although these attacks have long been known and described in detail. There is some noticeable progress in the configuration of the web server and its modules, but the tolerance to slow traffic is still, by default, high enough to perform a DoS attack. However, despite the possible stricter server settings, the created attack generators can adapt attacks and invoke the DoS state. The worst results were achieved by lighttpd 1.4.55 and Apache 2.4.17. Due to the lack of insufficient security against slow DoS, these servers were vulnerable to all tested attacks.

In the case of attacks on the HTTP/2 protocol, our generator confirms that the vulnerability of the Apache2 server has already been fixed in version 2.4.29, except the Slow Preface attack. This attack was successful on the Apache server in both tested versions. Other HTTP/2 attacks were successful only against the older version 2.4.17. We will verify the vulnerabilities of Microsoft IIS and lighttpd in future research once support for this protocol is fully implemented.

In conclusion, this paper confirms the importance of the security development and configuration adjustment of web servers to mitigate the mentioned attacks. We also strongly recommend the usage of additional protection, e.g., an intrusion prevention system. The contribution of our work is the creation of a universal generator of slow DoS attacks, which can be easily extended to new types of attacks. We have included the latest types of attacks in the generator, for which no tool is yet available. This generator allows testing web server vulnerabilities and the effectiveness of attack detection/mitigation implementations. Experimental testing of the generator showed the weaknesses of the current most used web servers. In this paper, we also described our proposal of attack prevention and detection mechanisms.

Our future goal is to complete the development of the generator, fix all bugs, and then publish it. In future work, we will also focus on testing the vulnerabilities of newer web server versions including the HTTP/2 implementation of lighttpd and the encrypted variant in Microsoft IIS. Thanks to the easy addition of other services to the experimental environment, we intend to test the vulnerabilities of other application services against these attacks. Our future work will also focus on the design, implementation, and testing of accurate detection and mitigation mechanisms.

## Figures and Tables

**Table 1:** Selected papers’ comparison.

Paper	Slowloris	Slow Next	Slowcomm	SlowDrop	Slow Read	Slow POST	Slow Preface	Slow Headers	Slow Settings	HTTP/2	Other DoS	Mitigation
[1]	**✓**	**✗**	**✗**	**✗**	**✓**	**✗**	**✗**	**✗**	**✗**	**✗**	**✓**	**✓**
[2]	**✓**	**✗**	**✗**	**✗**	**✓**	**✓**	**✗**	**✗**	**✗**	**✗**	**✓**	**✗**
[3]	**✓**	**✗**	**✗**	**✗**	**✗**	**✓**	**✗**	**✗**	**✗**	**✗**	**✓**	**✓**
[6]	**✓**	**✗**	**✗**	**✗**	**✗**	**✗**	**✗**	**✗**	**✗**	**✗**	**✓**	**✓**
[7]	**✓**	**✗**	**✗**	**✗**	**✗**	**✗**	**✗**	**✗**	**✗**	**✗**	**✓**	**✓**
[9]	**✗**	**✗**	**✓**	**✗**	**✗**	**✗**	**✗**	**✗**	**✗**	**✗**	**✓**	**✗**
[10]	**✓**	**✓**	**✗**	**✗**	**✗**	**✗**	**✗**	**✗**	**✗**	**✗**	**✓**	**✓**
[11]	**✓**	**✓**	**✗**	**✗**	**✓**	**✗**	**✗**	**✗**	**✗**	**✗**	**✗**	**✓**
[12]	**✓**	**✓**	**✗**	**✗**	**✓**	**✗**	**✗**	**✗**	**✗**	**✗**	**✓**	**✓**
[13]	**✗**	**✗**	**✗**	**✗**	**✗**	**✗**	**✗**	**✗**	**✗**	**✗**	**✗**	**✓**
[14]	**✓**	**✗**	**✗**	**✗**	**✗**	**✓**	**✗**	**✗**	**✗**	**✗**	**✗**	**✓**
[15]	**✓**	**✓**	**✓**	**✗**	**✗**	**✗**	**✗**	**✗**	**✗**	**✗**	**✗**	**✓**
[16]	**✗**	**✗**	**✗**	**✓**	**✗**	**✗**	**✗**	**✗**	**✗**	**✗**	**✗**	**✓**
[17]	**✗**	**✗**	**✗**	**✗**	**✗**	**✗**	**✗**	**✗**	**✗**	**✓**	**✗**	**✓**
[24]	**✗**	**✗**	**✗**	**✗**	**✗**	**✗**	**✗**	**✗**	**✗**	**✓**	**✗**	**✗**
[25]	**✗**	**✗**	**✗**	**✗**	**✗**	**✗**	**✗**	**✗**	**✗**	**✓**	**✗**	**✗**
[26]	**✗**	**✗**	**✗**	**✗**	**✗**	**✗**	**✗**	**✗**	**✗**	**✓**	**✗**	**✓**
This paper	**✓**	**✓**	**✓**	**✓**	**✓**	**✓**	**✓**	**✓**	**✓**	**✓**	**✗**	**✓**

**Table 2:** Summary of selected slow DoS attacks and their main pattern.

Attacks Targeting	Attack	Main Pattern of the Attack
HTTP/x	Slowloris	Missing terminating character \r\n\r\n
	Slow POST (R.U.D.Y.)	High value of Content-Length parameter
	Slow Read	Low value of window-size parameter
	SlowDrop	Response parts are randomly dropped
	Slowcomm	Incomplete request + requests generating
	Slow Next	Valid request and waiting
HTTP/2	Slow Read	SETTING_INITIAL_WINDOW_SIZE equals 0 WINDOW_UPDATE not sent
	Slow POST	HEADERS equals: END_STREAM: 1, END_HEADERS: 0
	Slow Preface	CONNECTION PREFACE form is set to: PRI*HTTP/2.0\r\n\r\nSM\r\n
	Slow Headers	For GET method: END_HEADERS: 0 and END_STREAM: 1 For POST method: END_HEADERS: 0 and END_STREAM: 0
	Slow Settings	Valid GET or POST request, Settings frame is not confirmed

**Table 3:** Comparison of the proposed generator from the Slowloris attack point of view.

Tool	Attack Specific Parameters						General Parameters								
	Fully Editable Header	Content-Length	User-Agent	Keep Alive Data	Method	Conn. per Seconds	Target IP	ARP	Total Number of Conn.	Time between Senders	Time between Waves	Starting Port	Port Step	Server URL	Bots Simulation
Proposed generator	**✓**	**✓** 42	**✓**	**✓** X-a: b	**✓** GET	**✗**	**✓**	**✓**	**✓** 500	**✓** 10 ms	**✓** 2 s	**✓** 5000	**✓** 1	**✓**	**✓**
slowhttptest	**✗**	**✓** 4096	**✗**	**✗**	**✓** GET	**✓** 50	**✓**	**✗**	**✓** 50	**✓** 10 s	**✗**	**✗**	**✗**	**✓**	**✗**
Slowloris	**✗**	**✗**	**✗**	**✗**	**✗**	**✗**	**✓**	**✗**	**✓** 1000	**✓** 5 s	**✗**	**✓** 80	**✗**	**✓**	**✗**
PyLoris	**✗**	**✗**	**✗**	**✗**	**✗**	**✗**	**✓**	**✗**	**✓**	**✓**	**✗**	**✓**	**✗**	**✓**	**✗**

**Table 4:** Comparison of the proposed generator from the Slow READ attack point of view.

Tool	Attack Specific Parameters				General Parameters								
	Window Size	Slow Read URL	Interval between Reads	Repeat Request	Target IP	ARP	Total Number of Conn.	Time between Senders	Time between Waves	Starting Port	Port Step	Server URL	Bots Simulation
Proposed generator	**✓** 10 B	**✓** /index.html	**✗**	**✓**	**✓**	**✓**	**✓** 500	**✓** 10 ms	**✓** 2 s	**✓** 5000	**✓** 1	**✓**	**✓**
slowhttptest	**✓** 5 B	**✗**	**✓** 1 s	**✓**	**✓**	**✗**	**✓** 50	**✓** 10 s	**✗**	**✗**	**✗**	**✓**	**✗**

**Table 5:** Comparison of the proposed generator from the Slow POST attack point of view.

Tool	Attack Specific Parameters				General Parameters								
	Fully Editable Header	Payload Size	Method	Tor	Target IP	ARP	Total Number of Conn.	Time between Senders	Time between Waves	Starting Port	Port step	Server URL	Bots Simulation
Proposed generator	**✓**	**✓** 1 GB	**✓** POST	**✗**	**✓**	**✓**	**✓** 500	**✓** 10 ms	**✓** 2 s	**✓** 5000	**✓** 1	**✓**	**✓**
slowhttptest	**✗**	**✗**	**✓** POST	**✗**	**✓**	**✗**	**✓** 50	**✓** 10 s	**✗**	**✗**	**✗**	**✓**	**✗**
R.U.D.Y.	**✗**	**✓** 1 Mb	**✓** POST	**✓**	**✓**	**✗**	**✓** 500	**✓** 5 s	**✗**	**✗**	**✗**	**✓**	**✗**

**Table 6:** Experimental network specification.

Type	Name	OS	CPU	RAM	IP	Web Server
Host machine	VMware Host	Windows 10 Pro 20H2	i7-7700 3.6 GHz	32 GB	10.0.0.2, 20.0.0.2	-
Guest machine	Router	Debian 10.8	4 cores	2 GB	10.0.0.1, 20.0.0.1	-
	Attacker	Kali Linux 2020.04	4 cores	2 GB	20.0.0.5	-
	Client	Debian 10.8	1 core	2 GB	20.0.0.3	-
	Web server 1	Debian 10.8	1 core	2 GB	10.0.0.3	Apache 2.4.17, lighttpd 1.4.55
	Web server 2	Debian 10.8	1 core	2 GB	10.0.0.4	Apache 2.4.26
	Web server 3	Windows 10 Pro 20H2	2 cores	2 GB	10.0.0.5	Microsoft IIS 10, Nginx 1.14.0

**Table 7:** Discovered vulnerabilities.

Attack	Apache 2.4.17	Apache 2.4.29	Nginx 1.14.0	Lighttpd 1.4.55	MS IIS 10.0
Slowloris	**✓**	**✓**	**✗**	**✓**	**✗**
Slow POST	**✓**	**✓**	**✗**	**✓**	**✗**
Slow Read	**✓**	**✓**	**✓**	**✓**	**✗**
SlowDrop	**✓**	**✓**	**✗**	**✓**	**✗**
Slowcomm	**✓**	**✓**	**✓**	**✓**	**✗**
Slow Next	**✓**	**✓**	**✗**	**✓**	**✗**
Slow POST (HTTP/2)	**✓**	**✗**	**✗**	-	-
Slow Read (HTTP/2)	**✓**	**✗**	**✗**	-	-
Slow Settings (HTTP/2)	**✓**	**✗**	**✗**	-	-
Slow Headers (HTTP/2)	**✓**	**✗**	**✗**	-	-
Slow Preface (HTTP/2)	**✓**	**✓**	**✗**	-	-

## Data Availability

The data presented in this study are available on request from the corresponding author. The license does not allow us to fully publish the source code.
